# Prediction model for recurrence probabilities after intravesical chemotherapy in patients with intermediate-risk non-muscle-invasive bladder cancer, including external validation

**DOI:** 10.1007/s00345-015-1598-0

**Published:** 2015-05-30

**Authors:** Rianne J. M. Lammers, Jan C. M. Hendriks, O. Rodriguez Faba Rodriguez Faba, Wim P. J. Witjes, Joan Palou, J. Alfred Witjes

**Affiliations:** Department of Urology, Radboud University Medical Centre, Geert Grooteplein Zuid 10 (659), P.O. Box 9101, 6500 HB Nijmegen, The Netherlands; Department for Health Evidence, Radboud University Medical Centre, Nijmegen, The Netherlands; Fundacio Puigvert, Oncology Urology, Barcelona, Spain; CuraTrial SMO & Research, Arnhem, The Netherlands

**Keywords:** Adjuvant chemotherapy, Intravesical administration, Prediction model, Recurrence, Urinary bladder neoplasm

## Abstract

**Purpose:**

To develop a model to predict recurrence for patients with intermediate-risk (IR) non-muscle-invasive bladder cancer (NMIBC) treated with intravesical chemotherapy which can be challenging because of the heterogeneous characteristics of these patients.

**Methods:**

Data from three Dutch trials were combined. Patients treated with intravesical chemotherapy with characteristics according to the IR definition of the EAU guideline 2013 were included. Uni- and multivariable Cox regression with selection methods were used to identify predictors of recurrence at 1, 2, and 5 years. An easy-readable table for recurrence probabilities was developed. An external validation was done using data from Spanish patients.

**Results:**

A total of 724 patients were available for analyses, of which 305 were primary patients. Recurrences occurred in 413 patients (57 %). History of recurrences, history of intravesical treatment, grade 2, multiple tumors, and adjuvant treatment with epirubicin were relevant predictors for recurrence-free survival with hazard ratios of 1.48, 1.38, 1.22, 1.56, and 1.27, respectively. A table for recurrence probabilities was developed using these five predictors. Based on the probability of recurrence, three risk groups were identified. Patients in each of the separate risk groups should be scheduled for less or more aggressive treatment. The model showed sufficient discrimination and good predictive accuracy. External validation showed good validity.

**Conclusion:**

In our model, we identified five relevant predictors for recurrence-free survival in IR-NMIBC patients treated with intravesical chemotherapy. These recurrence predictors allow the urologists to stratify patients in risk groups for recurrence that could help in deciding for an individualized treatment approach.

**Electronic supplementary material:**

The online version of this article (doi:10.1007/s00345-015-1598-0) contains supplementary material, which is available to authorized users.

## Introduction

Bladder cancer remains a common problem in the Western world [1]. Approximately 75–85 % of bladder cancer presents as non-muscle-invasive bladder cancer (NMIBC); the remaining patients have muscle-invasive disease (MIBC) [2].

Treatment of NMIBC consists of complete transurethral resection of the bladder tumor (TURBT), followed by a single immediate postoperative instillation (POI) with chemotherapy. Further treatment depends on patients’ and tumor characteristics. In the guidelines of the European Association of Urology (EAU), patients are divided into three risk groups [2] (Supplementary Table [ST] 1). This stratification is similar to that provided by the International Bladder Cancer Group (IBCG) [3] and is partially based on the well-known risk tables developed by the European Organization for Research and Treatment of Cancer (EORTC) [4].

In general, treatment advises for low-risk and high-risk groups are clearly stated in the guidelines, but treatment advises for intermediate-risk (IR) patients are less clear. This is an important lack of information as the IR group covers a large number of patients with heterogeneous characteristics, making selection of appropriate therapy challenging. Therefore, we identified predictors of recurrence and developed a prediction model for recurrence probabilities for IR-NMIBC patients treated with intravesical chemotherapy.

## Methods

Data of three prospective Dutch studies [5–7] were available for analyses, providing us with individual data of 2006 patients. Treatment and follow-up have been described in detail before [5–7] and can be found in ST2.

For the development of the prediction model, in this study, we included only patients with Ta G1/2 urothelial carcinoma *without* carcinoma in situ (CIS) and without the combination ‘multiple & recurrent & diameter >3 cm.’ This is consistent with the definition of IR group according to the EAU guideline [2]. All included patients received intravesical chemotherapy (either mitomycin C or epirubicin).

Primary outcome measurement was time to first recurrence (recurrence-free survival; RFS): time from randomization to the date of the first bladder recurrence.

### Statistical methods

First, baseline demographics of the selected Dutch patients are presented (*n* = 724).

Univariable and multivariable Cox regression with selection procedures and likelihood analyses were used for selecting independent variables for RFS. Smoking and tumor diameter were removed from analyses based on the number of missing data and the hazard ratios (HRs). For the final model, the adjusted HRs are presented, including the 1, 2, and 5 years probabilities for recurrence. To assess the model’s accuracy (discrimination), Harrell’s bias corrected concordance index (c-index) was calculated at 1, 2, and 5 years and models were refitted 200 times with bootstrap resampling techniques.

Three risk groups were constructed based on the risk profiles of the final model: <P33, P33–P66, and >P66. In addition, sensitivity, specificity, negative, and positive predictive value (NPV, PPV) were calculated for the minor risk group at 2 years, as most recurrences occurred within 2 years.

### External validation

Prospectively collected, independent, individual patient data provided by Fundacio Puigvert, Barcelona, Spain, were used to study the final prediction model (*n* = 137). However, this cohort included only data of primary patients. For the comparison, we used a subcohort of primary patients from the Dutch cohort (*n* = 305). The prediction model was applied to the data of these cohorts.

Statistical analyses were done with SPSS 20.0.0 for Windows (SPSS Inc., Chicago, Il, USA), in SAS 9.2 for Windows (SAS Institute Inc., Cary, NC, USA) and in R 2.2 for Windows.

## Results

### Demographics of Dutch cohort

Data of 724 Dutch patients met our inclusion criteria. The baseline demographics are presented in Table 1. The median follow-up was 29.6 months (range 2–239 months). A total of 413 patients (57 %) experienced a recurrence. As expected, only few patients progressed to MIBC (16 patients; 2.2 %), and therefore, we did not take progression into account as an outcome measurement.

**Table 1:** Demographics of the three cohorts

	Dutch cohort		Dutch subcohort of primary patient		Spanish cohort	
	Total (*n* = 724)	Recurrence (*n* = 413)	Total (*n* = 305)	Recurrence (*n* = 148)	Total (*n* = 137)	Recurrence (*n* = 79)
	*N* (%)/	*N* (%)/	*N* (%)/	*N* (%)/	*N* (%)/	*N* (%)/
	Median (range)	Median (range)	Median (range)	Median (range)	Median (range)	Median (range)
Age in years	67.5 (33–89)	66.9 (35–86)	65 (33–86)	64.9 (35–85)	69 (37–89)	69 (37–84)
Age classification						
≤66 year	335 (46)	195 (47)	161 (53)	78 (53)	61 (45)	37 (47)
>66 year	389 (54)	218 (53)	144 (47)	70 (47)	76 (55)	42 (53)
Gender						
Male	592 (82	337 (82)	246 (81)	117 (79)	109 (80)	61 (77)
Female	130 (18	74 (18)	59 (19)	31 (21)	28 (20)	18 (23)
Unknown	2	2	0	0	0	0
Primary or recurrent						
Primary	305 (42)	148 (36)	305 (100)	148 (100)	137 (100)	79 (100)
Recurrent	419 (58)	265 (64)	NA	NA	NA	NA
History of intravesical treatment						
No	580 (84)	309 (79)	305 (100)	148 (100)	317 (100)	79 (100)
Yes	115 (16)	83 (21)	NA	NA	NA	NA
Unknown	29		21			
Tumor grade						
G1	351 (48)	205 (50)	102 (33)	51 (35)	14 (10)	10 (13)
G2	373 (52)	208 (50)	203 (67)	97 (65)	123 (90)	69 (87)
Number of tumors						
Single	176 (25)	83 (20)	57 (19)	23 (16)	85 (63)	42 (54)
Multiple	542 (75)	324 (80)	248 (81)	125 (84)	50 (37)	36 (46
Unknown	6	6	0	0	2	1
Adjuvant treatment						
Mitomycin C	218 (30)	113 (27)	105 (34)	46 (31)	137 (100)	79 (100)
Epirubicin	506 (70)	300 (73)	200 (66)	102 (69)	0	0
Median follow-up in mo (range)	29.6 (2–239)	19.2 (2–239)	37.2 (2–128)	21 (2–128)	30.6 (3–112)	18.4 (3–97)

*CI* confidence interval, *HR* hazard ratio, *NA* not applicable for primary patients

### Recurrence

In Tables 2 and 3, we show the crude and adjusted HR with 95 % confidence interval (CI) of the clinicopathological characteristics using uni- and multivariable Cox regression. The following five variables were included in the final model: history of previous recurrences, history of intravesical treatment, tumor grade 2, multiple tumors, and adjuvant treatment with epirubicin, with HRs of 1.48, 1.38, 1.22, 1.56, and 1.27, respectively. As can be seen in Table 3, the HR, 95 % CI and *p* value of tumor grade and adjuvant treatment are 1.22 (95 % CI 0.99–1.51; *p* value 0.061) and 1.27 (95 % CI 1.00–1.62; *p* value 0.048), respectively. Although the statistical significance of these two variables is around 0.05, the five-variable model outperformed the three-variable model, i.e., the model without tumor grade and adjuvant treatment (likelihood ratio test, Chi-square = 17.0, *p* = 0.0002).

**Table 2:** Crude hazard ratios (HRs) and the 95 % confidence intervals (CI), using univariable Cox regression for time to recurrence for the three cohorts

	Dutch cohort (*n* = 724)			Dutch subcohort of primary patients (*n* = 305)			Spanish cohort (*n* = 137)		
	*N*	HR 95 % CI	*p* value	*N*	HR 95 % CI	*p* value	*N*	HR 95 % CI	*p* value
Age									
≤66 year	722	1 (ref)		305	1 (ref)		137	1 (ref)	
>66 year		1 (0.83–1.22)	0.978		1.05 (0.76–1.45)	0.774		0.92 (0.59–1.43)	0.703
Gender									
Male	720	1 (ref)		305	1 (ref)		137	1 (ref)	
Female		0.98 (0.76–1.26)	0.854		1.07 (0.72–1.58)	0.742		1.25 0.74–2.21)	0.407
Primary or recurrent									
Primary	722	1 (ref)		NA	NA NA	NA	NA	NA NA	NA
Recurrent		1.54 (1.25–1.88)	<0.001						
History of intravesical treatment									
No	694	1 (ref)		NA	NA NA	NA	NA	NA NA	NA
Yes		1.71 (1.34–2.18)	<0.001						
Tumor grade									
G1	722	1 (ref)		305	1 (ref)		137	1 (ref)	
G2		1.01 (0.84–1.23)	0.885		1.01 (0.72–1.42)	0.936		0.8 (0.41–1.56)	0.511
Tumor diameter	528			219			123		
≤30 mm		1 (ref)	0.365		1 (ref)			1 (ref)	
>30 mm		1.2 (0.81–1.79)			1.88 (1.20–2.96)	0.006		0.64 (0.34–1.19)	0.158
Number of tumors									
Single	716	1 (ref)		305	1 (ref)		135	1 (ref)	
Multiple		1.48 (1.16–1.88)	0.002		1.41 (0.90–2.21)	0.129		1.82 (1.16–2.84)	0.009
Smoking status	499			198			118		
No		1 (ref)	0.15		1 (ref)			1 (ref)	
Yes in past or now		1.28 (0.91–1.81)			1.68 (0.94–3.01)	0.08		0.79 (0.45–1.39)	0.411
Treatment									
Mitomycin C	722	1 (ref)	0.006	305	1 (ref)	0.11	–	–	–
Epirubicin		1.36 (1.09–1.69)			1.33 (0.94–1.89)				

*NA* not applicable to primary patients; – no data available due to 100 % treatment with mitomycin C

**Table 3:** Adjusted hazard ratios (HRs) and 95 % confidence intervals (CI), using multivariable Cox regression with selection procedures for time to recurrence for the three cohorts

	Dutch cohort (*n* = 724)		Dutch subcohort of primary patients (*n* = 305)		Spanish cohort (*n* = 137)	
	HR (95 % CI)	*p* value	HR (95 % CI)	*p* value	HR (95 % CI)	*p* value
Primary versus recurrent						
Primary	1 (ref)	0.001	NA (NA)	NA	NA (NA)	NA
Recurrent	1.48 (1.17–1.88)		NA (NA)		NA (NA)	
History of intravesical treatment						
None	1 (ref)	0.021	NA (NA)	NA	NA (NA)	NA
Yes	1.38 (1.05–1.80)		NA (NA)		NA (NA)	
Tumor grade						
G1	1 (ref)	0.061	1 (ref)	0.485	1 (ref)	0.861
G2	1.22 (0.99–1.51)		1.11 (0.78–1.58)		0.94 (0.48–1.85)	
No. of tumors						
Single	1 (ref)	0.001	1 (ref)	0.286	1 (ref)	0.011
Multiple	1.56 (1.20–2.01)		1.47 (0.92–12.34)		1.8 (1.15–2.84)	
Treatment						
Mitomycin C	1 (ref)	0.048	1 (ref)	0.278	–	–
Epirubicin	1.27 (1.00–1.62)		1.24 (0.84–1.84)		–	–

*NA* not applicable to primary patient; – no data available due to 100 % treatment with mitomycin C

### Prediction model

**Fig. 1:**
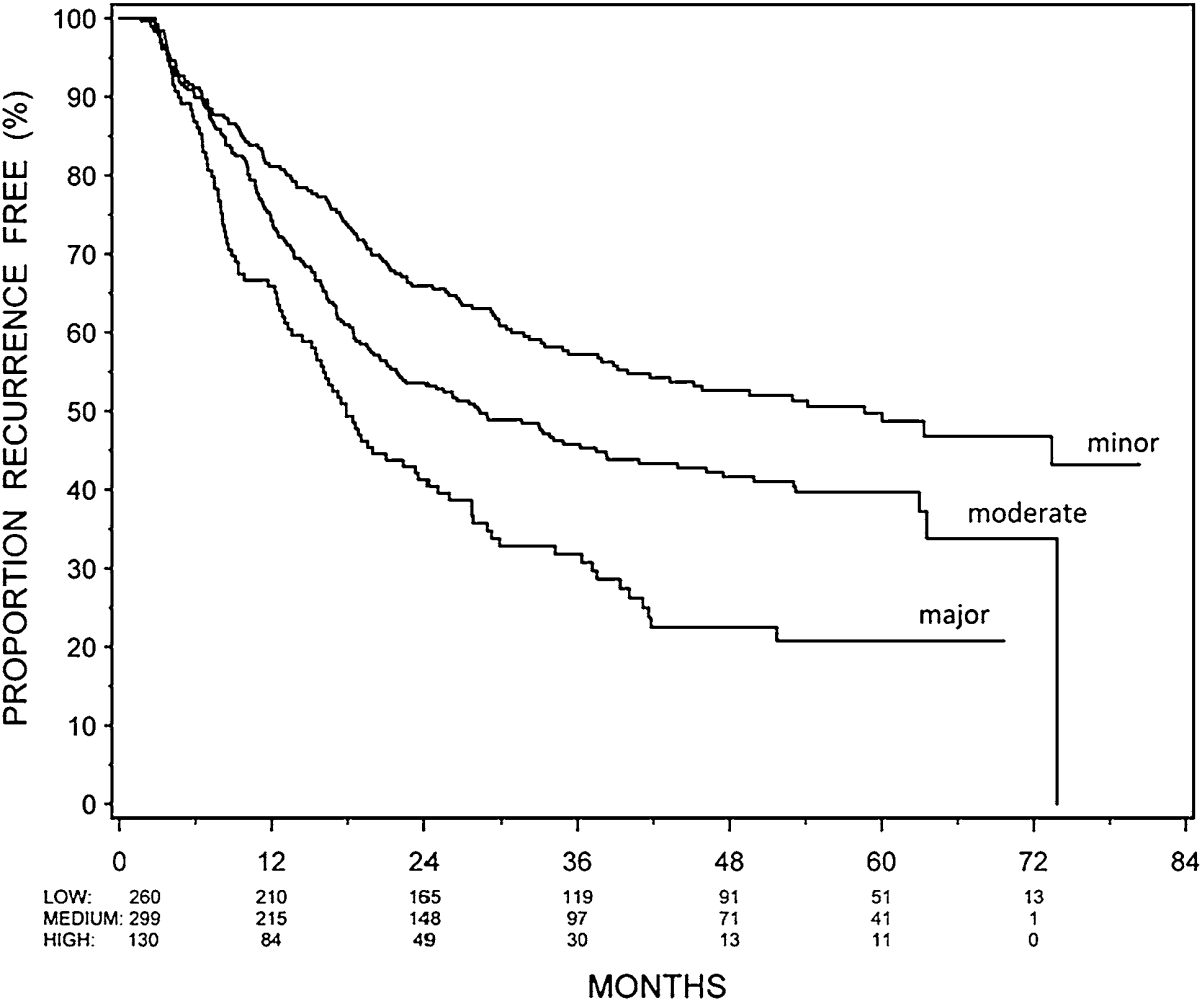
Proportion of recurrence-free patients per risk group

The recurrence probabilities of the final Cox regression model at 1, 2, and 5 years are presented in Table 4. The c-index for this RFS model was 0.60, 0.62, and 0.63 at year 1, 2, and 5, respectively. Three risk groups were constructed based on the risk profiles of the final model. The Kaplan–Meier curves of minor, moderate, and major risk based on the risk profiles of the final model are shown in Fig. 1. The model can distinguish clearly between recurrence outcomes, e.g., a patient with multiple G2 recurrences without previous treatment who received adjuvant treatment with epirubicin has, according to Table 4, 67 % chance of being recurrence free at 12 months, which is associated with major risk in Fig. 1. We calculated sensitivity, specificity, PPV, and NPV for minor risk patients at 2 years (ST3). PPV is 68.4 % and NPV is 65.2 %.

**Table 4:** Probabilities of being recurrence free at 1, 2, and 5 years in patients with non-muscle-invasive bladder cancer after treatment with intravesical chemotherapy

	Primary	Recurrent previous treatment	
		No	Yes
*1* *year*			
Grade 1			
Single			
Mitomycin C	89 (86–93)	85 (80–89)	79 (73–87)
Epirubicin	87 (82–91)	81 (76–86)	75 (76–83)
Multiple			
Mitomycin C	84 (79–89)	77 (71–83)	70 (62–79)
Epirubicin	80 (75–85)	72 (67–77)	63 (56–71)
Grade 2			
Single			
Mitomycin C	87 (83–91)	81 (76–87)	75 (68–84)
Epirubicin	84 (79–89)	77 (71–83)	70 (62–79)
Multiple			
Mitomycin C	81 (76–86)	73 (66–80)	64 (56–75)
Epirubicin	76 (71–81)	67 (60–74)	57 (49–67)
*2* *years*			
Grade 1			
Single			
Mitomycin C	80 (73–86)	71 (63–79)	62 (52–74)
Epirubicin	74 (67–82)	64 (57–73)	54 (44–67)
Multiple			
Mitomycin C	69 (62–77)	58 (50–68)	47 (37–60)
Epirubicin	63 (56–70)	50 (44–57)	39 (31–49)
Grade 2			
Single			
Mitomycin C	75 (69–82)	65 (57–75)	56 (45–69)
Epirubicin	69 (62–78)	58 (50–68)	48 (37–61)
Multiple			
Mitomycin C	64 (57–72)	52 (43–63)	40 (30–54)
Epirubicin	57 (50–64)	43 (36–52)	31 (23–42)
*5* *years*			
Grade 1			
Single			
Mitomycin C	68 (60–78)	57 (47–68)	46 (34–61)
Epirubicin	61 (52–72)	49 (40–59)	37 (27–52)
Multiple			
Mitomycin C	55 (47–65)	41 (32–53)	30 (20–44)
Epirubicin	47 (39–55)	33 (26–41)	21 (14–32)
Grade 2			
Single			
Mitomycin C	63 (54–72)	50 (40–62)	39 (28–54)
Epirubicin	55 (46–66)	41 (32–53)	30 (20–44)
Multiple			
Mitomycin C	48 (40–58)	34 (25–47)	23 (14–37)
Epirubicin	40 (33–48)	25 (18–35)	15 (9–25)

### Demographics of Spanish cohort and external validation

**Fig. 2:**
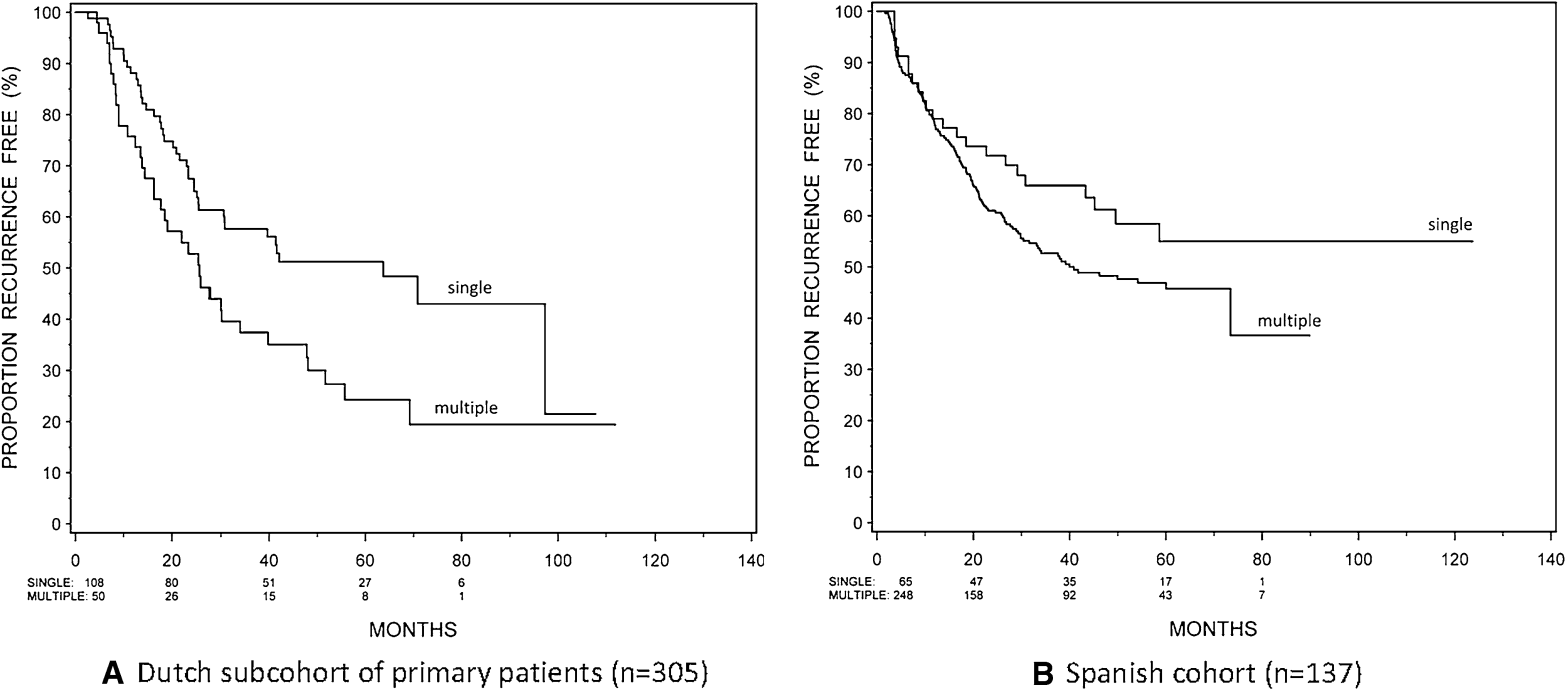
Two-year probability of recurrence free by number of tumors and by cohort, using Kaplan–Meier analyses. **a** Dutch subcohort of primary patients (*n* = 305), **b** Spanish cohort (*n* = 137)

For the external validation, a cohort of 137 Spanish patients was used (treatment and demographics can be found in ST2 and Table 1, respectively). It needs to be stressed that in the Spanish cohort, only ten patients had grade 1. Therefore, only the HR of the number of tumors was updated using the data of Dutch subcohort and the Spanish cohort. The HR of the number of tumors of 1.65 (95 % CI 1.28–2.00) in the combined cohorts (Dutch subcohort + Spanish cohort) was comparable to the development (Dutch) cohort: 1.56 (95 % CI 1.20–2.01). The associated Kaplan–Meier curves are shown in Fig. 2.

## Discussion

We present a study comparing recurrence outcome and treatment options for the heterogeneous spectrum of IR patients, and we propose a prediction model on recurrence probabilities with external validation. We found five relevant predictors for RFS: a history of recurrences, history of previous treatment, tumor grade 2, multiple tumors, and adjuvant treatment with epirubicin, with HRs of 1.48, 1.38, 1.22, 1.56, and 1.27, respectively. There is a huge difference between 1 and 5 years outcome, and between having none or all of the independent predictors (Table 4). We defined the IR group into three subgroups (minor, moderate, and major risk) in a way that each risk group needs to be considered for a less or more aggressive adjuvant treatment schedule or treatment type. The recurrence probabilities as predicted in Table 4 can be related to a risk group in Fig. 1.

The EORTC have developed risk tables based on a group of 2596 patients [4]. However, the EORTC risk tables have several limitations: 22 % of patients received no intravesical treatment at all; only 171 patients (7 %) received treatment with bacillus Calmette–Guerin (BCG) and none received BCG maintenance. Therefore, the EORTC risk tables could be interpreted as probabilities of the untreated natural history of the disease, especially for progression. Another well-known prediction model is the scoring model of Club Urologico Español de Tratamiento Oncologico (CUETO) [8]. Data of 1062 patients, all treated with BCG, were used to identify risk factors for recurrence and progression after BCG treatment. Several other prediction models have been developed for NMIBC [9–15], but none of them included solely patients who, according to the guidelines, should have been and in fact were treated with intravesical chemotherapy.

Recently, Kamat et al. [16] developed an algorithm specifically for IR patients based on the consensus of the IBCG. They consider tumor size, tumor multiplicity, timing and frequency of recurrences, and previous treatment to be key factors. Based on these key factors, they divide IR patients in three groups: low-risk patients, ‘true’ IR patients, and high-risk patients. Our analyses and model support these recommendations; only tumor size, which is also a significant predictor in the EORTC risk model [4], is of no influence in our model, and tumor grade is not considered to be a key factor by the IBCG.

Concerning tumor size, Kamat et al. [16] do mention that the well-known cutoff of 3 cm might be no longer relevant as the number of patients with large tumors is very low, which we could confirm (in the Dutch cohort only 6 %). Within the IBCG, it was suggested to further study a new cutoff of 1 cm. When analyzing this cutoff in our cohort, no statistical significant influence on recurrence outcome was seen (*p* = 0.480), but this might be due to the high number of missing data, which is a limitation of our study.

We found that tumor diameter had many missing data and was no statistically significant predictor for RFS in the complete cohort. This is clearly different from other prediction models including the EORTC risk model [4], but our group of patients all received adjuvant intravesical chemotherapy and are therefore not comparable with, e.g., the EORTC risk model patients.

The term ‘low grade’ is based on the WHO 2004 grading system, which was not yet available during the inclusion period of the three Dutch studies. Therefore, we considered G1 and G2 tumors to be low grade, but G2 tumors are a mixture of low-grade and high-grade tumors. According to Chen et al. [17], approximately 80 % of the G2 tumors are low grade. Thus, in this study, we could have misclassified 75 patients, and consequently, these patients could be treated insufficiently with subsequently more recurrences.

The c-index of our model was 0.60, 0.62 and 0.63 at year 1, 2, and 5, respectively. This is comparable to the c-index for recurrence probabilities of the EORTC risk Tables (0.66 both at 1 and 5 years) and that of the CUETO scoring model (0.64 both at 1 and 5 years) [4, 8]. However, the clinical relevance of the c-index is doubtful and there is no consensus how high the c-index should be to make a model clinically relevant.

For a more practical approach, based on their risk factors, we divided the patients in three subgroups: minor, moderate and major risk. As can be seen in Fig. 1, this subdivision is clearly related to recurrence outcome, and thus, the major risk group could be considered for more aggressive treatment and the minor risk group for less aggressive treatment. The relevance of this subdivision is also reflected in the predictive accuracy of our model (ST3). For treatment options, the NPV and PPV are more important than sensitivity and specificity, as this is associated with under- and overtreatment. Compared to the EORTC and CUETO, our model is clearly better in preventing overtreatment in minor risk patients as PPV is much higher (68 % versus 21 and 24 %), but NPV is somewhat lower (65 versus 94 and 92 %) which, however, is less of a problem in minor risk patients [18]. Additionally, the external validation shows very good overlap in HR. However, as the Spanish cohort only included primary patients, it is in fact a partial external validation. Nevertheless, as agreement between the Dutch subcohort and the Spanish cohort is high, one could hypothesize that these results could be extrapolated to the total model. An external validation with primary and recurrent patients is needed to confirm our results.

Limitations of this study are the long inclusion period, the missing data, and the differences with the current standard of treatment including the quality of TURBT due to, e.g., the introduction of re-TURBT, the introduction of fluorescence cystoscopy, and the lack of immediate POIs in most patients (only 23 % of patients received immediate POI). On the other hand, fluorescence cystoscopy is most useful in CIS, but these patients were excluded in our analyses. In the Dutch cohort, no re-TURBT was done, but this is not always necessary in IR patients. Another limitation is the variability in adjuvant treatment, including the dose, the concentration of chemotherapy used, and the treatment schedule which might have influenced the outcome. Yet, the median number of instillations received was 10, and only 2.1 % of patients received less than six instillations. Furthermore, both European and American guidelines do not recommend specific chemotherapy schedules [2, 19].

## Conclusion

We developed a risk table for IR-NMIBC patients treated with intravesical chemotherapy including five relevant predictors of RFS: history of recurrences, history of intravesical treatment, grade 2, multiple tumors and adjuvant treatment with epirubicin. These individual predictors were used to subdivide IR patients into three risk groups, which is related to recurrence outcome. With this subcategorization, the urologist together with the patient can choose for an individualized treatment approach.

## Electronic supplementary material

Supplementary material 1 (DOCX 23 kb)

## Abbreviations

BCG: Bacillus Calmette–Guerin
CI: Confidence interval
CIS: Carcinoma in situ
CUETO: Club Urologico Espanol de Tratamiento Oncologica
C-index: Harrell’s bias corrected concordance index
EAU: European association of urology
EORTC: European Organization for Research and Treatment of Cancer
HR: Hazard ratio
IBCG: International bladder cancer group
IR: Intermediate risk
MIBC: Muscle-invasive bladder cancer
NMIBC: Non-muscle-invasive bladder cancer
NPV: Negative predictive value
POI: Postoperative instillation
PPV: Positive predictive value
RFS: Recurrence-free survival
ST: Supplementary table
TURBT: Transurethral resection of bladder tumor
WHO: World Health Organization
