# Supplementary material for: Prediction model for recurrence probabilities after intravesical chemotherapy in patients with intermediate-risk non-muscle-invasive bladder cancer, including external validation
Source: World J Urol. 2015 May 30;34:173–80. doi: 10.1007/s00345-015-1598-0 (PMC4729802; doi:10.1007/s00345-015-1598-0)
Supplement: Supplementary file 1 — Supplementary material 1 (DOCX 23 kb) [file 345_2015_1598_MOESM1_ESM.docx]

**Supplementary tables**

| **Supplementary table 1.** Risk group stratification according to EAU guideline 2013 [[1](#_ENREF_1)] | |
| --- | --- |
| Risk group | Definition |
| Low risk tumors | Primary, solitary, Ta, LG/G1, <3 cm, no CIS |
| IR tumors | All tumors not defined in the two adjacent categories (between the category of low and high risk) |
| High risk tumors | Any of the following:   - T1 tumor - HG/G3 tumor - CIS - Multiple and recurrent and large (>3 cm) Ta, G1/G2 tumors (all conditions must be presented in this point) |
| CIS = carcinoma in situ; HG = high grade; LG = low grade. | |

1. Babjuk M, Burger M, Zigeuner R, Shariat SF, van Rhijn BW, Comperat E, Sylvester RJ, Kaasinen E, Bohle A, Palou Redorta J, Roupret M (2013) EAU Guidelines on Non-Muscle-invasive Urothelial Carcinoma of the Bladder: Update 2013. European urology 64 (4):639-653. doi:10.1016/j.eururo.2013.06.003

| **Supplementary table 2.** Treatment details | | | | |
| --- | --- | --- | --- | --- |
|  | Dutch cohort | | | Spanish cohort |
|  | MMC versus BCG-Tice versus BCG-RIVM [[2](#_ENREF_2)] | Epirubicin trial [[3](#_ENREF_3)] | KLH versus MMC [[4](#_ENREF_4)] |  |
| Study design | Multicenter RCT | Multicenter RCT | Multicenter RCT | Fundacio Puigvert, Barcelona, Spain |
| Inclusion period | April 1987 – December 1990 | April 1998 - April 2004 | July 2003 – November 2007 | March 2000–December 2009 |
| Patients | Primary and recurrent pTa-T1, including CIS | Intermediate- and high risk, no CIS | Intermediate- and high risk, no CIS | All new NMIBC patients |
| Treatment schedule | All: complete TURBT  MMC: 4 weekly instillations, 6 monthly instillations, and in case of persistent or recurrent disease 3 additionally monthly instillations.  BCG: 6 weekly instillations, in case of persistent or recurrent disease 6 additional weekly instillations | All: complete TURBT.  Group 1: 4 weekly  and 5 monthly instillations (standard schedule),  Group 2: standard schedule + 1 instillation <48h after TURBT.  Group 3: standard schedule + 2 instillations at 9 and 12 months | All: complete TURBT  MMC: 4 weekly instillations, 5 monthly instillations, 2 additional instillations at 9 at 12 months.  KLH: pre-immunization with intracutaneous KLH, 6 weekly instillations and 10 monthly instillations | All: complete TURBT including random biopsies, direct postoperative instillation with MMC, 4 weekly instillations and 3 monthly instillations with MMC. |
| Dose | 30mg MMC in 50mL saline | 50mg epirubicin in 50mL saline | 40mg MMC in 50mL saline | 40mg MMC in 50mL saline |
| BCG = bacillus Calmette Guerin; CIS = carcinoma in situ; KLH = keyhole limpet hemocyanin; MMC = mitomycin C; RCT = randomized clinical trial; TURBT = transurethral resection of bladder tumor. | | | | |

2. Vegt PD, Witjes JA, Witjes WP, Doesburg WH, Debruyne FM, van der Meijden AP (1995) A randomized study of intravesical mitomycin C, bacillus Calmette-Guerin Tice and bacillus Calmette-Guerin RIVM treatment in pTa-pT1 papillary carcinoma and carcinoma in situ of the bladder. JUrol 153 (3 Pt 2):929-933

3. Hendricksen K, Witjes WP, Idema JG, Kums JJ, van Vierssen Trip OB, de Bruin MJ, Vergunst H, Caris CT, Janzing-Pastors MH, Witjes JA (2008) Comparison of three schedules of intravesical epirubicin in patients with non-muscle-invasive bladder cancer. EurUrol 53 (5):984-991

4. Lammers RJ, Witjes WP, Janzing-Pastors MH, Caris CT, Witjes JA (2012) Intracutaneous and Intravesical Immunotherapy With Keyhole Limpet Hemocyanin Compared With Intravesical Mitomycin in Patients With Non-Muscle-Invasive Bladder Cancer: Results From a Prospective Randomized Phase III Trial. Journal of clinical oncology : official journal of the American Society of Clinical Oncology. doi:10.1200/jco.2011.39.2936

| **Supplementary table 3.** Sensitivity, specificity, positive and negative predictive value for minor risk patients at 2 years | | |
| --- | --- | --- |
|  | n/N | % |
| Patients who had a recurrence and are moderate or major risk (sensitivity) | 210/298 | 70.5% |
| Patients who had no recurrence and are minor risk (specificity) | 165/362 | 45.6% |
| Patients with moderate or major risk who recurred (positive predictive value) | 210/307 | 68.4% |
| Patients with minor risk who remained recurrence-free (negative predictive value) | 135/253 | 65.2% |
